# Immunological Approaches to Biomass Characterization and Utilization

**DOI:** 10.3389/fbioe.2015.00173

**Published:** 2015-10-28

**Authors:** Sivakumar Pattathil, Utku Avci, Tiantian Zhang, Claudia L. Cardenas, Michael G. Hahn

**Affiliations:** ^1^Complex Carbohydrate Research Center, University of Georgia, Athens, GA, USA; ^2^Oak Ridge National Laboratory, BioEnergy Science Center (BESC), Oak Ridge, TN, USA

**Keywords:** glycome profiling, immunolocalization, cell walls, biomass, antibodies

## Abstract

Plant biomass is the major renewable feedstock resource for sustainable generation of alternative transportation fuels to replace fossil carbon-derived fuels. Lignocellulosic cell walls are the principal component of plant biomass. Hence, a detailed understanding of plant cell wall structure and biosynthesis is an important aspect of bioenergy research. Cell walls are dynamic in their composition and structure, varying considerably among different organs, cells, and developmental stages of plants. Hence, tools are needed that are highly efficient and broadly applicable at various levels of plant biomass-based bioenergy research. The use of plant cell wall glycan-directed probes has seen increasing use over the past decade as an excellent approach for the detailed characterization of cell walls. Large collections of such probes directed against most major cell wall glycans are currently available worldwide. The largest and most diverse set of such probes consists of cell wall glycan-directed monoclonal antibodies (McAbs). These McAbs can be used as immunological probes to comprehensively monitor the overall presence, extractability, and distribution patterns among cell types of most major cell wall glycan epitopes using two mutually complementary immunological approaches, glycome profiling (an *in vitro* platform) and immunolocalization (an *in situ* platform). Significant progress has been made recently in the overall understanding of plant biomass structure, composition, and modifications with the application of these immunological approaches. This review focuses on such advances made in plant biomass analyses across diverse areas of bioenergy research.

## Introduction

### Complexity and Dynamics of Plant Cell Walls Constituting Biomass

Plant biomass, the prime feedstock for lignocellulosic biofuel production, constitutes the principal sustainable resource for renewable bioenergy. Identifying the optimal plant biomass types that are most suitable for biofuel production and optimizing their downstream processing and utilization are at the forefront of modern-day lignocellulosic feedstock research. The focus of much of this research is the examination of diverse classes of plants for their potential as cost-effective and sustainable raw materials for biofuel production. For example, biomass materials originating from classes of plants ranging from herbaceous dicots (e.g., alfalfa), woody dicots (e.g., poplar), perennial monocots (e.g., *Agave* spp.), herbaceous monocots (e.g., grasses such as *Miscanthus*, sugarcane, and switchgrass), and woody gymnosperms (e.g., pines) are regarded as potentially promising resources for biofuel production (Galbe and Zacchi, 2007; Gomez et al., 2008; Somerville et al., 2010).

Cell walls constitute the major part of plant biomass, and physicochemical features of these cell walls vary among biomass materials from diverse plant classes (Pauly and Keegstra, 2008; Popper, 2008; Fangel et al., 2012). For example, cell walls from grass biomass have distinct structural and compositional features [with a higher abundance of glucuronoarabinoxylans and the presence of mixed-linkage glucans (Vogel, 2008)] that are quite different from those of highly lignified woody biomass (Studer et al., 2011) or herbaceous dicot biomass (Burton et al., 2010; Liepman et al., 2010). Even within a plant, the structure and composition of cell walls can vary significantly depending on the cell types, organs, age, developmental stage, and growth environment (Freshour et al., 1996; Knox, 2008). These cell wall variations are the result of differences in the relative proportions and structural dynamics that occur among the major cell wall polymers, which include (but are not limited to) cellulose, hemicelluloses, pectic polysaccharides, and lignin (Pauly and Keegstra, 2008). Several structural models for plant cell walls have been proposed and published (McNeil et al., 1984; McCann and Roberts, 1991; Carpita and Gibeaut, 1993; Carpita, 1996; Cosgrove, 1997; Somerville et al., 2004; Loqué et al., 2015); all of these models focus on the primary wall. To our knowledge, no model has been proposed for secondary plant cell walls, which constitute the bulk of the biomass used for bioenergy production. In vascular plants, non-glycan components such as lignin (especially in secondary cell wall-containing tissues such as sclerenchyma and xylem cells) are important for optimal growth and development of plants by playing important roles in maintaining cell wall integrity to optimally facilitate water transportation, rendering mechanical support and defense against pathogens (Weng and Chapple, 2010; Voxeur et al., 2015). A high abundance of lignin in cell walls is regarded as disadvantageous for biomass utilization for biofuel production as it contributes significantly to recalcitrance. Transgenic plants that are genetically modified for reduced lignin biosynthesis have been shown to exhibit reduced recalcitrance properties (Chen and Dixon, 2007; Pattathil et al., 2012b). The abundance of diverse potential plant biomass feedstocks that are available to be studied and the aforementioned variations among the cell walls constituting them pose a major challenge in lignocellulosic bioenergy research.

Research on the structure, function, and biosynthesis of plant cell walls has received new impetus with advances in genome sequencing that have made available, for the first time, whole genomes from diverse plant families. Thus, complete genomes have been sequenced for plants from diverse phylogenetic classes including both herbaceous [e.g., *Arabidopsis* (The Arabidopsis Genome Initiative, 2000); *Medicago* (Young et al., 2011)] and woody dicots [e.g., *Populus* (Tuskan et al., 2006)] and monocotyledonous grasses [e.g., maize (Schnable et al., 2009), rice (Goff et al., 2002; Yu et al., 2002), and brachypodium (The International Brachypodium Initiative, 2010)]. The availability of these genome sequences has, in turn, dramatically expanded experimental access to genes and gene families involved in plant primary and secondary cell wall biosynthesis and modification. Functional characterization of cell wall-related genes and the proteins that they encode, combined with expanded research on cell wall deconstruction, have dramatically enhanced our understanding of wall features important for biomass utilization.

### Genetic Approaches to Studies of Cell Walls with Impacts on Lignocellulosic Bioenergy Research

Cell walls are known for their innate resistance to degradation and specifically to the breakdown of their complex polysaccharides into simpler fermentable sugars that can be utilized for microbial production of biofuels. This property of plant cell walls is referred to as “recalcitrance” (Himmel et al., 2007; Fu et al., 2011). Cell wall recalcitrance has been identified as the most well-documented challenge that limits biomass conversion into sustainable and cost-effective biofuel production (Himmel et al., 2007; Pauly and Keegstra, 2008; Scheller et al., 2010). Hence, identifying cell wall components that affect recalcitrance has been an important target of lignocellulosic bioenergy research (Ferraz et al., 2014). A number of plant cell wall polymers, including lignin, hemicelluloses, and pectic polysaccharides, have been shown to contribute to cell wall recalcitrance (Mohnen et al., 2008; Fu et al., 2011; Studer et al., 2011; Pattathil et al., 2012b).

Most of the studies directed toward overcoming recalcitrance focus on genetically modifying plants by specifically targeting genes involved in the biosynthesis or modification of wall polymers (Chen and Dixon, 2007; Mohnen et al., 2008; Fu et al., 2011; Studer et al., 2011; Pattathil et al., 2012b) with the objective of generating a viable, sustainable biomass crop that synthesizes cell walls with reduced recalcitrance. Identification of target genes for reducing recalcitrance has relied largely on model plant systems, particularly *Arabidopsis*, and then to transfer that information to biofuel crops. This has been particularly successful for genes and pathways that participate directly or indirectly in secondary cell wall biosynthesis and development. Secondary walls constitute the bulk of most biofuel feedstocks and thus become a main target for genetic modification (Chundawat et al., 2011; Yang et al., 2013). Secondary wall synthetic genes that have been investigated in this way include, for example, several genes that are involved in cellulose [such as various *CesA* genes (Joshi et al., 2004, 2011; Taylor et al., 2004; Brown et al., 2005; Ye et al., 2006)] and xylan biosynthesis [*IRX8* (Brown et al., 2005; Ye et al., 2006; Peña et al., 2007; Oikawa et al., 2010; Liang et al., 2013), *IRX9* (Brown et al., 2005; Lee et al., 2007, 2011a; Peña et al., 2007; Oikawa et al., 2010; Liang et al., 2013), *IRX9L* (Oikawa et al., 2010; Wu et al., 2010), *IRX14* (Oikawa et al., 2010; Wu et al., 2010; Lee et al., 2011a), *IRX14L* (Wu et al., 2010; Lee et al., 2011a), *IRX15* (Brown et al., 2011), and *IRX15L* (Brown et al., 2011)] in dicots. In addition, a number of transcription factors including plant-specific NAC-domain transcription factors [*SND1*, *NST1*, *VND6*, and *VND7* in *Arabidopsis* (Kubo et al., 2005; Zhong et al., 2006, 2007b)], WRKY transcription factors [in *Medicago* and *Arabidopsis* (Wang et al., 2010; Wang and Dixon, 2012)], and MYB transcription factors [*MYB83* (McCarthy et al., 2009) and *MYB46* (Zhong et al., 2007a) in *Arabidopsis*] with potential involvement in secondary wall biosynthesis and development have been functionally characterized. Examples of the successful transfer of insights gained in model dicots to studies of orthologous genes in monocots include investigations of rice *IRX* orthologs involved in xylan biosynthesis and secondary wall formation (Oikawa et al., 2010) and experiments on transcription factors controlling secondary wall formation in several grasses (Handakumbura and Hazen, 2012; Shen et al., 2013; Valdivia et al., 2013). These molecular genetic approaches toward understanding and manipulating cell wall-related genes for biofuel feedstock improvement would be assisted by improved methods for rapidly identifying and characterizing the effects of genetic changes on cell wall components.

### Need for Efficient Tools for Plant Cell Wall/Biomass Analyses

The structural complexity of plant cell walls, regardless of their origin, is challenging to analyze, particularly in a high-throughput manner. To date, most of the plant cell wall analytical platforms have been based on the preparation of cell wall materials and/or extracts that are selectively enriched for particular wall polysaccharides, followed by colorimetric assays (Selvendran and O’Neill, 1987), chemical derivatizations coupled with gas chromatography (Albersheim et al., 1967; Sweet et al., 1974, 1975a,b), mass spectroscopy (Lerouxel et al., 2002), and nuclear magnetic resonance spectroscopy (NMR) (Peña et al., 2008) to gain compositional and structural information about those polysaccharides. Some of these methods have been adapted for biomass analytics [see, for review, Sluiter et al. (2010)]. Overall, these tools have allowed extensive progress in delineating basic structural features of diverse classes of plant cell wall polysaccharides. However, these experimental approaches for plant cell wall/biomass analysis are time-consuming, require specialized and, in some cases, expensive equipment, are low in throughput, and usually provide information only about a single polysaccharide of specific interest. However, given the number of wall components that have already been shown to influence cell wall recalcitrance, and the complex and heterogeneous nature of cell wall components in diverse plants, it is desirable to have additional tools, particularly those with higher throughput and the capability to monitor a broad spectrum of wall polymers. Over the past 10 years, immunological approaches for plant cell wall and biomass analyses have emerged as tools that are broadly applicable to multiple aspects of interests to the biofuel research community, including characterization of genetically altered plant feedstocks, investigations of the effects of diverse biomass pretreatment processes, and the effects of enzymatic or microbial deconstruction of cell walls. In the following sections, we review applications of two immunological tools for studies on plant biomass that employ a comprehensive collection of plant cell wall glycan-directed probes.

## Probes for Biomass Analyses

Currently, well-characterized cell wall-directed probes range from small molecules (Wallace and Anderson, 2012) to larger proteinaceous probes such as carbohydrate-binding modules (CBMs) and monoclonal or polyclonal antibodies (Knox, 2008; Pattathil et al., 2010; Lee et al., 2011b). In this review, we will focus on the latter cell wall-directed probes.

### Glycan-Directed Probes

#### Monoclonal Antibodies

**Figure 1:**
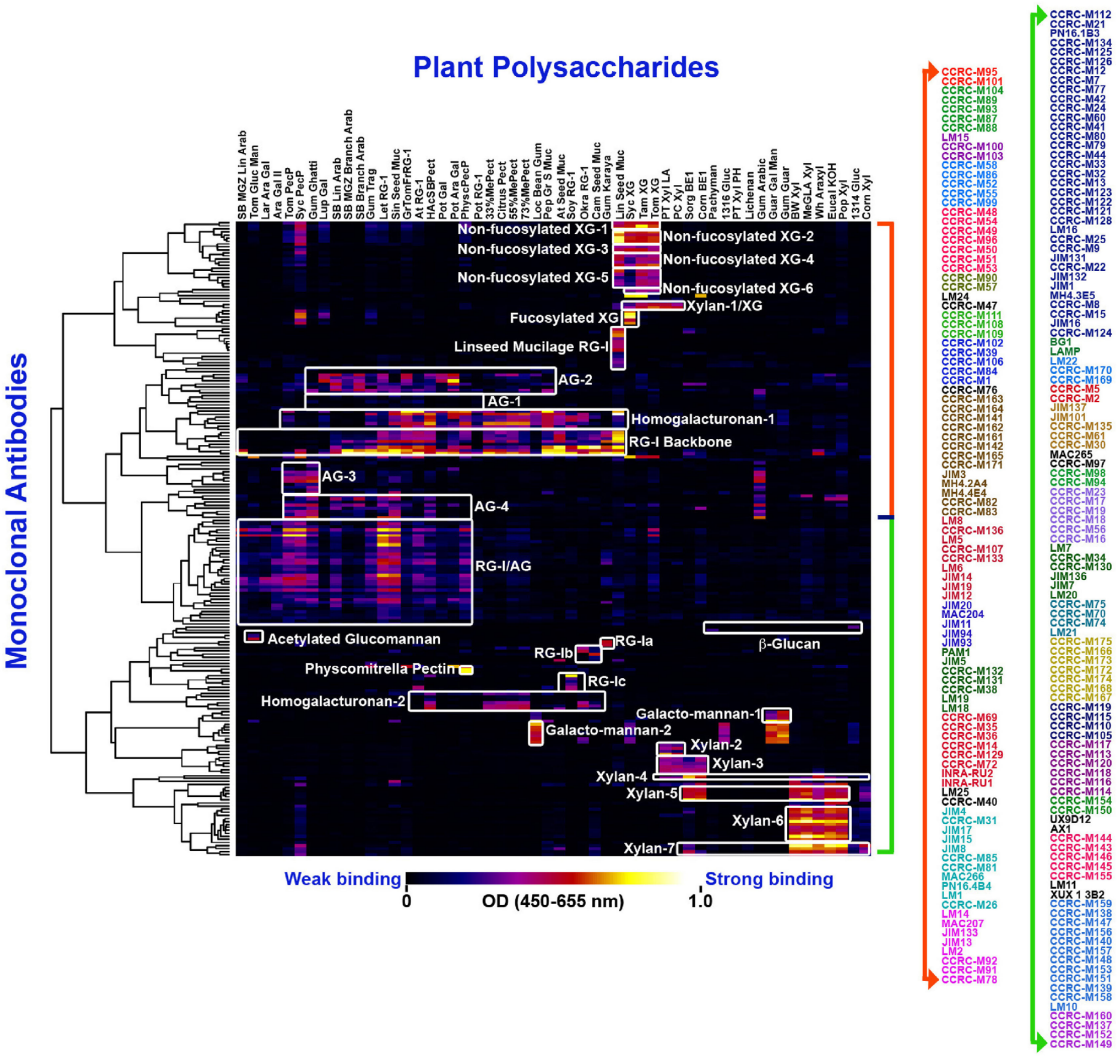
**Current worldwide collection of plant cell wall glycan-directed McAbs: the entire collection of ~210 McAbs was ELISA-screened against a panel of 54 structurally known plant cell wall carbohydrate preparations (Pattathil et al., 2010) and they were clustered to 31 groups (as depicted by the white blocks) based on their binding specificities**. The binding strengths are depicted in a dark blue–red–bright yellow color scheme where maximum and no binding are denoted by bright yellow color and dark blue colors, respectively. The names of individual McAbs are denoted on the right hand panel in different colors denoting 31 groups.

Plant cell wall glycan-directed monoclonal antibodies (McAbs) are among the most commonly used probes for plant cell wall analyses. McAbs, commonly available as hybridoma culture supernatants, are monospecific probes that recognize specific glycan sub-structures (epitopes) present in plant polysaccharides (Knox, 2008; Pattathil et al., 2010). McAbs have several advantages that make them particularly suited for use as glycan-directed probes. First, since each antibody is the product of a single clonal cell line, each McAb is by definition monospecific with regard to the epitope that is recognized. This is important for studies of glycans, whose structures are frequently repetitive and whose substructures can be found in multiple macromolecular contexts (e.g., arabinogalactan epitopes present on glycoproteins and on rhamnogalacturonan I). The monospecific nature of McAbs also means that, in theory, the binding specificity of the antibody can be determined unambiguously, although this is still difficult for glycan-directed antibodies given the complexity of plant cell wall glycan structures. McAbs also typically bind to their epitopes with high affinity (*K*_d_ ~10^−6^ M), which makes them very sensitive reagents for detecting and quantitating molecules to which they bind. Finally, another significant advantage with McAbs is that their supply is not limited, as cell lines producing them can be cryopreserved indefinitely (some hybridoma lines whose plant glycan-directed antibodies are frequently used today were generated more than 20 years ago) and can be regrown at any time to produce additional McAb, which retains the binding selectivity and affinity of the original McAb, as needed in any quantities required. Currently, a worldwide collection of over 200 McAbs (Pattathil et al., 2010, 2012a) exists (Figure 1) that encompasses antibodies recognizing diverse structural features of most major non-cellulosic cell wall glycans, including arabinogalactans, xyloglucans, xylans, mannans, homogalacturonans, and rhamnogalacturonan I. So far, McAbs that bind reliably and specifically to rhamnogalacturonan II have not been reported. The available plant glycan-directed McAbs can be obtained from several stock centers (see Table 1) or from the individual research laboratories that generated them. A listing of the McAbs currently available is not practical here. The reader is referred to a plant cell wall McAb database, Wall*Mab*DB,^1^ where detailed descriptions of most of the currently available plant glycan-directed McAbs, including immunogen, antibody isotype, and epitope structure (to the extent known), can be obtained.

**Table 1:** **List of major CBMs currently used for plant cell wall analyses**.

Group	Protein	Enzyme	Organism	Type	Reference
A. Cellulose-binding group	CBM1	Cellulase	*Trichoderma reesei*	Crystalline cellulose	Reinikainen et al. (1992)
	CBM2a	Xylanase 10A	*Cybister japonicus*	Crystalline cellulose	Bolam et al. (1998)
	CBM3a	Scaffoldin	*Clostridium thermocellum*	Crystalline cellulose	Tormo et al. (1996)
	CBM10	Xylanase 10A	*Cybister japonicas*	Crystalline cellulose	Gill et al. (1999)
	CBM4-1	Cellulase 9B	*Cellulomonas fimi*	Amorphous cellulose	Tomme et al. (1996)
	CBM17	Cellulase 5A	*Clostridium cellulovorans*	Amorphous cellulose	Boraston et al. (2000)
	CBM28	Cellulase 5A	*Bacillus* sp. no. 1139	Amorphous cellulose	Boraston et al. (2002)
	CBM9-2	Xylanase 10A	*Thermotoga maritima*	The ends of cellulose chain	Boraston et al. (2001)
B. Xylan-binding group	CBM2b-1-2	Xylanase 11A	*Cellulomonas fimi*	Both decorated and unsubstituted xylan	Bolam et al. (2001)
	CBM4-2	Xylanase 10A	*Rhodothermus marinus*	Both decorated and unsubstituted xylan	Abou Hachem et al. (2000)
	CBM6	Xylanase 11A	*Clostridium thermocellum*	Both decorated and unsubstituted xylan	Czjzek et al. (2001)
	CBM15	Xylanase 10C	*Cybister japonicus*	Both decorated and unsubstituted xylan	Szabó et al. (2001)
	CBM22-2	Xylanse 10B	*Clostridium thermocellum*	both decorated and unsubstituted xylan	Charnock et al. (2000)
	CBM35	Arabino-furano-sidase 62A	*Cybister japonicus*	Unsubstituted xylan	Bolam et al. (2004)
C. Mannan-binding group	CBM27 (TmMan5)	Mannanase 5C	*Thermotoga maritima*	Mannan	Filonova et al. (2007) and Zhang et al. (2014)
	CBM35 (Cjman5C)	Mannanase 5C	*Cybister japonicus*	Mannan	Filonova et al. (2007) and Zhang et al. (2014)
D. Xyloglucan-binding group	CBMXG34	Modified xylanase 10A	*Rhodothermus marinus*	Non-fucosylated xyloglucan	Gunnarsson et al. (2006)
	CBMXG34/1-X	Modified xylanase 10A	*Rhodothermus marinus*	Non-fucosylated xyloglucan	von Schantz et al. (2009)
	CBMXG34/2-VI	Modified xylanase 10A	*Rhodothermus marinus*	Non-fucosylated xyloglucan	von Schantz et al. (2009)
	CBMXG35	Modified xylanase 10A	*Rhodothermus marinus*	Non-fucosylated xyloglucan	Gunnarsson et al. (2006)
E. Pectic galactan-binding group	TmCBM61	GH53 endo-β-1,4-galac-tanase	*Thermotoga maritima*	β-1,4-galactan	Cid et al. (2010)

Early studies in our laboratory screened 130 of the plant glycan-directed McAbs available at the time for their binding specificity to 54 structurally characterized polysaccharide preparations from diverse plants (Pattathil et al., 2010). Hierarchical clustering analyses of the resultant binding response data resolved the McAbs into 19 antibody clades based on their binding specificities to the 54 plant glycans tested (Pattathil et al., 2010). A more recent study that included almost all available plant glycan-directed McAbs further resolved the antibody collection into about 31 clades of McAbs (Pattathil et al., 2012a). Figure 1 shows the data from most recent screening studies employing ~210 plant glycan-directed McAbs. While these broad specificity screens provide considerable information about the binding specificities of the McAbs in the collection, they do not provide complete detailed epitope information for the antibodies. Such detailed epitope characterization studies require the availability of purified, structurally characterized oligosaccharide fragments and/or purified and characterized glycosylhydrolases capable of selectively attacking epitope structures. To date, a relatively small number of plant glycan-directed McAbs have had their epitopes characterized in detail using these resources (Meikle et al., 1991, 1994; Puhlmann et al., 1994; Steffan et al., 1995; Willats et al., 2000a; Clausen et al., 2003, 2004; McCartney et al., 2005; Verhertbruggen et al., 2009; Marcus et al., 2010; Ralet et al., 2010; Pedersen et al., 2012; Schmidt et al., 2015). Recent advances in methods for immobilization of oligosaccharides on solid surfaces (Fukui et al., 2002; Wang et al., 2002; Willats et al., 2002; Blixt et al., 2004; Pedersen et al., 2012) is facilitating such epitope characterization studies, but the bottleneck remains the availability of comprehensive sets of purified, well-characterized plant glycan-related oligosaccharides.

#### Carbohydrate-Binding Modules

Carbohydrate-binding modules are another set of proteinaceous probes that have been used to study plant polysaccharide localization patterns *in vivo* (Knox, 2008). CBMs are amino acid sequences that are contiguous with the catalytic domain in a carbohydrate-active enzyme and are capable of binding to a carbohydrate structural domain (McCartney et al., 2006; Knox, 2008). CBMs have been shown to enhance the efficiency of cell wall hydrolytic enzymes by facilitating sustained and close contact between their associated catalytic modules and targeted substrates (Boraston et al., 2004; Zhang et al., 2014). Although CBMs have been known to occur in several plant enzymes, most CBMs that are used as probes for cell wall glycans are microbial in origin (Boraston et al., 2004; Shoseyov et al., 2006). CBMs, in contrast to the antibody probes described above, are relatively easy to prepare, given that their gene/protein sequences are known (McCann and Knox, 2011). CBMs have been classified into 71 sequence-based families.^2^ CBMs from approximately half of these families have been shown to bind to diverse plant cell wall polysaccharides, including cellulose (Blake et al., 2006), mannans (Filonova et al., 2007), xylans (McCartney et al., 2006), and most recently, the galactan side chains of rhamnogalacturonan I (Cid et al., 2010). Protein engineering of a xylan-binding CBM using random mutagenesis, phage-display technology, and affinity maturation has been employed to generate xyloglucan-specific CBMs (Gunnarsson et al., 2006; von Schantz et al., 2009, 2012), showing that it is possible to generate CBMs with new and heretofore unseen specificities.

Carbohydrate-binding modules that have been used to detect cellulose, xylan, mannan, xyloglucan, and pectic galactans in plant cells and tissues, together with information about their origins, are listed in Table 1. Binding of various CBMs is usually assessed by an indirect triple-labeling immunofluorescence procedure (His-tagged CBM, anti-His mouse-Ig, and anti-mouse Ig fluorescein isothiocyanate) in plant tissue sections (Knox, 2008; Hervé et al., 2010), which is slightly more complicated than the double-labeling procedure used with McAbs (Avci et al., 2012). The binding specificities exhibited by the CBMs enlarge the suite of probes available for biomass analyses, given that at least some of them bind to carbohydrate structures, such as cellulose substructures, for which no McAbs probes have been developed to date. Additional advantages of the CBMs are the availability of their gene and protein sequences and the wealth of structural information, including in many instances X-ray crystal structures, about their binding sites. Potential disadvantages of CBMs are their typically lower affinity for their ligands and the lower selectivity of their binding sites compared with McAb probes. Nonetheless, CBMs are useful probes for analyzing biomass.

### Immunological Probes Against Lignin

Lignins are phenylpropanoid polymers comprising 5–30% of biomass weight and have been considered as important sources of renewable aromatics (McKendry, 2002). Lignin composition and structure vary considerably depending on the plant species and on the cell type where lignins are deposited (Ruel et al., 1994; Donaldson, 2001). For example, in gymnosperms, lignins are mainly composed of guaiacyl units, whereas in angiosperms, lignins are formed by guaiacyl and syringyl units (Donaldson, 2001). In angiosperms, the guaiacyl-containing lignins are located mainly in secondary cell walls of vessels while syringyl-containing lignins are found on fibers (Ruel et al., 1994; Joseleau et al., 2004; Patten et al., 2010). Lignin composition and localization are also affected by pretreatment strategies aimed at removing lignin from biomass. For example, potassium permanganate labeling and electron microscopy studies revealed morphological alterations in *Zea mays* lignins subjected to different thermochemical pretreatments (Donohoe et al., 2008).

Lignin is most frequently visualized in plant tissue sections using selectively reactive histochemical stains such as phloroglucinol–HCl and Mäule reaction that can distinguish guaiacyl-enriched from syringyl-enriched cell wall regions (Patten et al., 2010). Although the various histochemical lignin stains provide general information about the localization of different lignin types, they cannot provide detailed information about specific lignin substructures; this would require more highly selective probes.

Given the structural complexity and variability of lignin, several laboratories have undertaken the development of immunological probes for lignins and/or lignin substructures. Much of the early work in this area focused on the production of polyclonal antisera. Thus, polyclonal antisera were raised against synthetic dehydrogenative polymers (DHPs) prepared from the appropriate *p*-hydroxycinnamic alcohols [*p*-hydroxyphenylpropane (H), guaiacyl (G), or syringyl (S), or mixtures of these] (Ruel et al., 1994; Joseleau et al., 2004). These polyclonal sera showed specificity toward the DHPs used to generate them. Other laboratories have generated polyclonal sera against milled wood lignin (Kim and Koh, 1997) or model compounds based on lignin substructures (Kukkola et al., 2003, 2004). The main difficulty with these polyclonal sera is that they are in limited supply, and many of these antisera are no longer available. Thus, new immunizations must be carried out, with uncertain outcomes with regard to the ability to reproduce the specificity of the original antisera; a fundamental problem with polyclonal antisera. In an effort to overcome this limited supply issue, two lignin-related model compounds, dehydrodiconiferyl alcohol and pinoresinol, were used to generate McAbs against these two lignin dimers (Kiyoto et al., 2013); supplies of these antibodies should not be limited. The antibody directed to dehydrodiconiferyl alcohol (KM1) displayed specificity toward a dehydrodiconiferyl alcohol 8-5′ model compound, whereas the antibody directed against pinoresinol (KM2) responded to two 8-8′ model compounds, pinoresinol and syrangaresinol. This recent development suggests that it will be possible, in principle, to generate specific McAbs against diverse lignin substructures. The number and diversity of lignin-directed McAbs will need to be increased in order to fully exploit these probes for greater insights into lignin structural diversity, localization patterns, and integration into the plant cell wall.

## Two Major Approaches for McAb/CBM-Based Analyses of Plant Biomass

The use of McAb/CBM probes to define the localization of plant cell wall components has a long history. These probes have been used in basic plant cell wall research to study the effects of mutations in wall-related genes on plant cell wall structure and composition, to study changes in plant cell walls during growth, development, and differentiation, and to study changes in plant cell walls that result from environmental and pathogenic influences. A comprehensive review of this literature is beyond the scope of this minireview and the reader is referred to several recent reviews to gain an overview of this literature (Knox, 1997, 2008; Willats et al., 2000b; Lee et al., 2011b; McCann and Knox, 2011). The use of McAb probes, in particular, is rapidly expanding due to the recent dramatic increase in the number and diversity of plant cell wall-directed antibodies (Pattathil et al., 2010) and the availability of more detailed information about the epitopes recognized by these McAbs (Pedersen et al., 2012; Schmidt et al., 2015).

We will concentrate here on an overview of recent studies that have taken advantage of the availability of the comprehensive collection of cell wall-directed McAb/CBM probes for studying plant biomass of interest as possible lignocellulosic feedstocks for biofuel production. These studies have focused on using these probes to understand the effects of genetic modification on biomass recalcitrance, to study the effects of different pretreatment regimes on biomass digestibility, and to study how microbes being considered for consolidated bioprocessing deconstruct plant biomass. Two complementary experimental approaches have been principally employed in these studies, namely, glycome profiling (Moller et al., 2007, 2008; Pattathil et al., 2012a) and immunolocalization (Avci et al., 2012). The following sections provide an overview of the studies with bioenergy implications done to date using these approaches.

### Studies Using Glycome Profiling

Glycome profiling involves the sequential extraction of insoluble cell wall/biomass samples with a series of reagents of increasing harshness and then screening the extracted cell wall materials with McAbs to determine which cell wall polymers are released in which extract. Thus, this experimental method provides two pieces of important information: (1) it provides detailed information about the composition of the biomass/cell walls; and (2) it provides information on how tightly the various wall components that can be detected are linked into the wall structure. The method is limited by the number of probes (McAbs, CBMs, etc.) used in the screen and the extent to which they are able to recognize the full breadth of wall components released by the extractive reagents. The substantial increase in number and diversity of cell wall probes over the past 10 years has dramatically improved the power and versatility of glycome profiling as a technique for rapid screening of cell wall/biomass samples.

The versatility of glycome profiling is also limited by the ability to immobilize the extracted wall components to a solid support. Diverse solid supports have been used, including nitrocellulose (Moller et al., 2007, 2008), glass slides (Pedersen et al., 2012), and multiwell plastic plates (Pattathil et al., 2012a). All of these suffer the limitation that most low-molecular-weight cell wall components that might be released in the wall extracts, especially low-molecular-weight glycans, do not bind to the solid supports without modification and therefore cannot be assayed by glycome profiling. The lower limit of the glycan size that will adhere has not been definitively determined, but is greater than 10 kDa (Pattathil et al., 2010).

The choice of extractive reagents that have been used for glycome profiling analyses has varied, as has their order. However, typically, the extractive reagents are used in order of increasing severity. Thus, relatively mild reagents, such as CDTA (Moller et al., 2007) or oxalate (Pattathil et al., 2012a), are used first, typically extracting primarily arabinogalactans and pectins. Harsher base extractions then follow, in which primarily hemicelluloses (e.g., xylans and xyloglucans) are extracted (Moller et al., 2007; Pattathil et al., 2012a). For samples that contain significant amounts of lignin, which is the case for most biomass samples of interest to the biofuel industry, an acidic chlorite extraction (Ahlgren and Goring, 1971; Selvendran et al., 1975) is used to degrade the lignin and release lignin-associated wall glycans; this chlorite extraction has most frequently been used after the first base extractions (Pattathil et al., 2012a) but has also been used as the first extraction step (de Souza et al., 2013). None of the extraction sequences used to date yield exclusively one kind of polymer in any given extract, an indication that each wall glycan exists as different subclasses that vary in their extent of cross-linking/interactions within the wall. Ultimately, the choice of extraction reagents and their order depends on the individual investigator and the specific research questions under investigation.

Two approaches for glycome profiling of plant biomass/cell wall samples have been described. The first, termed comprehensive microarray polymer profiling (CoMPP), is a dot blot-based assay system utilizing nitrocellulose as the solid support (Moller et al., 2007, 2008) and typically employs ~20 glycan-directed probes for screening of three sequential extracts [CDTA (50 mM), 4M NaOH, and Cadoxen (33%; v/v)] prepared from plant cell walls. The number of glycan-directed probes that could be used in CoMPP can readily be expanded. An alternative, ELISA-based approach, termed glycome profiling, uses 384-well microtiter plates as the solid support, and uses a broadly diverse toolkit of 155 plant glycan-directed McAbs (Pattathil et al., 2012a) to screen sets of sequentially prepared plant biomass/cell wall extracts [typically, oxalate (50 mM), carbonate (50 mM), 1M KOH, 4M KOH, acidified chlorite, and 4M KOH post-chlorite]. The use of a suite of 155 McAbs ensures a wide-ranging coverage of multiple structural features on most of the major non-cellulosic plant wall glycans (Zhu et al., 2010; Pattathil et al., 2012a). The ELISA-based approach used in glycome profiling lends itself to facile automation and quantitation of antibody binding, hence substantially increasing the throughput of the analyses.

Glycome profiling has seen broad application to diverse experimental approaches in lignocellulosic bioenergy research, including analyzing cell walls from native/genetically modified, variously pretreated, and microbially/enzymatically converted plant biomass (DeMartini et al., 2011; Duceppe et al., 2012; Lee et al., 2012; Tan et al., 2013; Biswal et al., 2015; de Souza et al., 2015; Pattathil et al., 2015; Trajano et al., 2015). Both CoMPP and glycome profiling have been used to undertake comparative glycomics of plant cell wall samples originating from diverse plant phylogenies (Popper et al., 2011; Sørensen et al., 2011; Duceppe et al., 2012; Kulkarni et al., 2012). Examples of such analyses applied to questions related to bioenergy research include a recent study assessing the genetic variability of cell wall degradability of a selected number of *Medicago* cultivars with superior saccharification properties (Duceppe et al., 2012) and an examination of five grass species that revealed commonalities and variations in the overall wall composition and extractability of epitopes among these grasses (Kulkarni et al., 2012). Glycome profiling has also been employed as an effective tool for analyzing cell walls from biomass crops that are genetically modified with the aim of reducing recalcitrance. Examples include examination of the effects on recalcitrance of mutations in lignin biosynthesis in alfalfa [*cad1* (cinnamyl alcohol dehydrogenase 1) (Zhao et al., 2013) and *hct* (hydroxycinnamoyl CoA:shikimate hydroxycinnamoyl transferase) (Pattathil et al., 2012b)] and overexpression of the secondary wall-related transcription factor, PvMYB4 in switchgrass (Shen et al., 2013).

Analyses using cell wall-directed probes have allowed the rapid identification and monitoring of structural and compositional alterations that occur in plant biomass under various regimes of pretreatments (Alonso-Simón et al., 2010; DeMartini et al., 2011; Li et al., 2014; Socha et al., 2014; Pattathil et al., 2015; Trajano et al., 2015). Studies on hydrothermally pretreated wheat straw using CoMPP showed that severe pretreatment regimes induce significant alterations in wheat straw biomass, including reduction in various hemicellulose and mixed-linkage glucan epitopes (Alonso-Simón et al., 2010). In a more recent study, glycome profiling of poplar biomass subjected to low, medium, and severe hydrothermal pretreatment regimes demonstrated that a series of structural and compositional changes occur in poplar cell walls during this pretreatment, including the rapid disruption of lignin–polysaccharide interactions even under mild conditions, with a concomitant loss of pectins and arabinogalactans, followed by significant removal of hemicellulose (xylans and xyloglucans) (DeMartini et al., 2011). The major inference from this study was that lignin content *per se* does not affect recalcitrance; instead, it is the associations/cross-links between polymers, for example, between lignin and various polysaccharides, within cell walls that play a larger role (DeMartini et al., 2011). Glycome profiling has also been used to examine the effects of other types of pretreatment regimes such as Ammonia Fiber Expansion (AFEX™), alkaline hydrogen peroxide (AHP), and various types of ionic liquids (ILs) on the composition and extractability of wall glycan epitopes in biomass samples from diverse bioenergy crop plants (Li et al., 2014; Socha et al., 2014; Pattathil et al., 2015). These studies demonstrate that, unlike hydrothermal pretreatment, these three types of pretreatment, in general, cause loosening of specific classes of non-cellulosic glycans from plant cell walls, thereby contributing to the reduced recalcitrance exhibited by the pretreated biomasses. Conclusions from these studies contribute significantly to a deeper understanding of pretreatment mechanisms and ultimately will enable optimization of biomass pretreatment regimes and perhaps further downstream utilization processes for biomass from different plant feedstocks.

Glycome profiling has also been used to identify cell wall components that affect biomass recalcitrance. A recent study examined poplar and switchgrass biomass subjected to different pretreatments and correlated pretreatment-induced changes in the biomass with recalcitrance properties of the treated biomass samples (DeMartini et al., 2013). A set of samples with varying composition and structure was generated from native poplar and switchgrass biomass via defined chemical and enzymatic extraction. Subsequently, glycome profiling of the extracts was employed to delineate which wall components were removed and residual solid pretreated biomass samples were analyzed for their recalcitrance features. Major conclusions from this study are that pretreatment regimes affect distinct biomass samples differently and that the most important contributors to recalcitrance vary depending on the biomass. Thus, lignin content appears to play an important role in biomass recalcitrance particularly in woody biomass such as poplar (as they contain higher levels of lignin). However, subclasses of hemicellulose were key recalcitrance-causing factors in grasses such as switchgrass. These results may have important implications for the biofuel industry as they suggest that biomass-processing conditions may have to be tailored to the biomass being used as the feedstock for biofuel generation (DeMartini et al., 2013).

Another bioenergy-related area that has benefited from the use of plant cell wall glycan-directed probes is research into how microbes, particularly those being selected for biomass deconstruction, degrade plant biomass during culture. Such knowledge will be useful for bioengineering microbes for better biomass conversion. An analysis of biological conversion of unpretreated wild-type sorghum and various *brown midrib* (*bmr*) lines by *Clostridium phytofermentans* examined variations in extractable polysaccharide epitopes of the cell-wall fractions in detail using glycome profiling (Lee et al., 2012). The conclusions were that the loosely integrated xylans and pectins are the primary polysaccharide targets of *C. phytofermentans* and that these are more accessible in the *bmr* mutants than in the wild-type plants (Lee et al., 2012). In another study, an anaerobic thermophilic bacterium, *Caldicellulosiruptor bescii*, was shown to solubilize both lignin and carbohydrates simultaneously in swichgrass biomass at high temperature (Kataeva et al., 2013). Further studies with *C. bescii* demonstrated that deletion of a cluster of genes encoding pectic-degrading enzymes in this organism compromised the ability of *C. bescii* to grow on diverse biomass samples (Chung et al., 2014). A comparative analysis of hemicellulose utilization potentials of *Clostridium clariflavum* and *Clostridium thermocellum* strains demonstrated that *C. clariflavum* strains were better able to grow on untreated switchgrass biomass and degraded easily extractable xylans more readily than do *C. thermocellum* strains (Izquierdo et al., 2014). In all of these studies, glycome profiling proved to be a very effective tool for understanding what was happening to the biomass during culture with the microbes. Studies of this kind provide information about the mode of action of microbial strains on plant biomass, thus identifying wall components that are resistant/recalcitrant to microbial actions.

### Studies Using Immunolocalization

Immunolocalization techniques use fixed and embedded (generally in plastic resins) biomass samples (Knox, 1997; Lee et al., 2011b). Primary probes (polyclonal antibodies, McAbs, and CBMs) are applied on semithin sections followed by probing with a fluorescently tagged secondary antibody that allows visualization of glycan epitope localization/distribution under a fluorescent microscope (Avci et al., 2012; Lee and Knox, 2014). This approach for biomass analyses provides information regarding the distribution of cell wall glycans at the cellular and subcellular levels.

A handful of studies thus far have employed this technique in the context of bioenergy research for analyses of cell walls in wall biosynthetic mutants and in pretreated biomass. Examination of *Arabidopsis* and *Medicago* mutants in which a WRKY transcription factor was knocked out revealed secondary cell wall thickening in pith cells caused by ectopic deposition of lignin, xylan, and cellulose. In the *Arabidopsis* mutant, this ectopic secondary wall formation resulted in an approximately 50% increase in biomass density in stem tissue (Yu et al., 2014). The use of three xylan-directed McAbs and a cellulose-directed CBM were instrumental in proving the ectopic deposition of these cell wall glycans in pith cells. In another recent study, the use of two xylan-directed CBMs (CBM2b-1-2 and CBM35 recognizing different degrees of methyl esterification on xylan) on the *Arabidopsis gxmt-1* mutant demonstrated a reduction of 4-*O*-methyl esterification of xylans (up to 75% as detected by chemical analyses) with a concomitant reduction in the recalcitrance of mutant walls (Urbanowicz et al., 2012). Additional studies also implicate the importance of secondary wall xylan for cell wall recalcitrance. Restoration of xylan synthesis in xylan-deficient mutants, as documented using xylan-directed McAbs, could, in some cases, yield plants with reduced xylan deposition compared with wild-type plants, but with normal growth habits and decreased recalcitrance (Petersen et al., 2012). Likewise, reduction of xylan in rice culm cell walls yielded plants with slightly lower stature, but with reduced recalcitrance (Chen et al., 2013).

Plant glycan-directed probes (McAbs and CBMs) can also be used to study the distribution patterns of glycan epitopes in plant biomass after diverse pretreatments used to reduce cell wall recalcitrance. One example of such a study is the demonstration that increasingly harsh hydrothermal pretreatments lead to an increased loss of various hemicellulosic, pectic, and cellulosic epitopes in cell walls of the pretreated tissues (DeMartini et al., 2011). The effects of other pretreatment methods (Alonso-Simón et al., 2010; DeMartini et al., 2013; Li et al., 2014; Socha et al., 2014; Pattathil et al., 2015; Trajano et al., 2015) on glycan epitope distribution patterns have not yet been carried out. Such information could be potentially useful to chemical engineers for the optimization of pretreatment conditions to enable optimal biomass conversion.

Immunolocalization studies have documented lignin distribution patterns in plant cell walls that may be relevant to bioenergy research. For instance, cell wall ultrastructure studies using three polyclonal antisera against DHPs allowed visualization of where these types of lignin-related polymers were located in cells of *Zea mays* L. (Joseleau and Ruel, 1997), *Arabidopsis thaliana*, *Nicotiana tabacum*, and *Populus tremula* (Ruel et al., 2002). These studies showed that H-DHPs were present in cell corners and middle lamella, whereas G-DHPs and G/S-DHPs were mainly present in secondary cell walls. The syringylpropane DHP epitope was visualized mainly in the S2 layer of secondary cell walls of *A. thaliana*, *N. tabacum*, and *P. tremula* (Joseleau et al., 2004). Recently, immunogold labeling analyses using KM1 and KM2 demonstrated the presence of 8-5′ and 8-8′ linked structures, respectively, on either developed xylem or phloem fibers of *Chamaecyparis obtusa* (Kiyoto et al., 2013). It will likely be informative to use these and other lignin-directed probes to monitor lignin distribution patterns in biomass that has been subjected to various pretreatment regimes and/or subjected to microbial degradation in the context of biomass conversion.

### Concluding Remarks

The application of high affinity, highly selective molecular probes against plant cell wall polymers clearly has high potential to provide complementary and supplementary data to existing chemical and biochemical analyses for studies on plant biomass structure and conversion. The number and diversity of McAb and CBM probes directed against plant polymers is now sufficiently large that these probes can provide extensive information about cell wall composition and structure in native and pretreated or microbially digested biomass. We have reviewed two main approaches using these probes for biomass characterization and conversion studies. Both glycome profiling/CoMPP and immunolocalization methods provide distinct but complementary information about the cell walls that constitute the bulk of plant biomass. Glycome profiling and CoMPP provide extensive information about the epitope composition and epitope extractability of polymers present in the biomass. Histochemical approaches using these probes provide valuable information about the spatial distribution of wall epitopes at all levels of organization, ranging from whole plants, to organs, to tissues, to cells, and even to individual cell walls and cell wall domains.

It is important to recognize several attributes of molecular probes directed against cell wall glycan epitopes, in particular, when interpreting the results of experiments. Both McAbs and CBMs are epitope-directed probes, that is, they specifically recognize particular structural motifs. Hence, glycan-directed McAbs and CBMs may not always be polymer-specific, in as much as glycan structures are frequently present in multiple molecular contexts within plant cell walls (e.g., arabinogalactan epitopes present on both polypeptide and polysaccharide backbones). Hence, positive binding of a McAb or CBM probe does not necessarily infer the presence of a particular cell wall glycan polymer. Likewise, the absence of binding of a given McAb or CBM does not unambiguously infer the absence of the glycan detected by this probe; the epitope may be absent or chemically modified (e.g., acetylated or methylated) such that the probe does not bind, but the polymer may still be present (Avci et al., 2012). Furthermore, plant glycans exist as families of polymers, whose epitope composition may not be uniform among all family members. Thus, a single McAb or CBM probe may not bind to all members of a polymer family, and it is therefore advisable to use multiple probes against diverse epitopes on a particular glycan to obtain a comprehensive picture of its abundance either in cell wall extracts or in histochemical localization studies. The size and diversity of the McAb/CBM collections now make such comprehensive studies possible.

Glycome profiling and CoMPP are dependent on the successful immobilization of cell wall-derived molecules to solid supports (e.g., plastic ELISA plates or nitrocellulose). Cell wall glycans with lower molecular masses (less than 20 kDa) have been found not to adhere reliably to the plates (Pattathil et al., 2010, 2012a). Hence, using glycome profiling as a tool to gather information regarding low-molecular-weight cell wall glycans is not advisable unless alternative strategies are employed to ensure adherence of these molecules to a solid support [e.g., covalent attachment directly to the solid support (Schmidt et al., 2015) or to a protein carrier that adheres to the solid support (Pedersen et al., 2012)]. Both glycome profiling and CoMPP also rely on chemical/enzymatic extractions of biomass/cell wall samples. Such extractions are rarely complete or quantitative and thus absolute quantitation of epitope composition in biomass/cell wall samples using these approaches is problematic. Thus, these approaches are best used as initial broad glycome characterization screens, particularly in comparative studies (e.g., mutant vs. wild-type and pretreated vs. untreated) where they provide valuable information regarding changes in the cell wall/biomass samples as a result of a particular experimental manipulation. In histochemical studies, the embedding medium used may influence the results of labeling experiments; in our laboratory, we have found LR White to give the most consistent results with both McAb and CBM probes (Avci et al., 2012).

## Future Perspectives

The molecular probe toolkits (McAb and CBM) currently available provide an invaluable resource for plant biomass analyses of relevance to bioenergy research and biomass conversion process development. In spite of the number and diversity of the probes currently available, there is still a need for additional probes against structural features not encompassed by the binding specificities of the probes currently available. Thus, additional probes against lignin substructures, rhamnogalacturonan II, and cellulose would further enhance the utility of the probe toolkit. In addition, coverage by the current probe collection of the epitope diversity for some cell wall glycans (e.g., mannans, glucomannans, and galactomannans) is limited. Finally, there remains a need to obtain more detailed information regarding the binding specificities of many of the molecular probes in the toolkit; about one third of the glycan-directed McAbs have had their epitope specificities characterized in detail. Efforts are underway in multiple laboratories to address these needs. Thus, we can look forward to an enhanced toolkit of probes against plant cell wall polymers in the future.

## Conflict of Interest Statement

The authors declare that the research was conducted in the absence of any commercial or financial relationships that could be construed as a potential conflict of interest.

## Footnotes

^1^http://www.wallmabdb.net.

^2^http://www.cazy.org/Carbohydrate-Binding-Modules.html.
